# Efficacy and safety of topical human keratinocyte growth factor-2 for dry eye disease: evidence from *in vivo* studies

**DOI:** 10.3389/fphar.2026.1800966

**Published:** 2026-03-25

**Authors:** Juan Li, Qiqi Wu, Zeyuan Wu, Weijun Xu, Panyu Zhang, Le Li, Bingjie Yu, Qi Hui, Kwang Youl Lee, Xiaojie Wang

**Affiliations:** 1 School of Pharmaceutical Sciences, Wenzhou Medical University, Wenzhou, Zhejiang, China; 2 College of Pharmacy and Research Institute of Drug Development, Chonnam National University, Gwangju, Republic of Korea

**Keywords:** dry eye disease, human keratinocyte growth factor 2, ocular surface, safety pharmacology, therapeutic efficacy

## Abstract

**Purpose:**

This study aimed to assess the therapeutic efficacy and safety pharmacology of human keratinocyte growth factor-2 (hKGF-2) eye drops for dry eye disease (DED).

**Methods:**

We assessed the primary pharmacodynamics of hKGF-2 in two animal models of DED: the benzalkonium chloride (BAC)-induced rabbit model and the scopolamine (SCOP)-induced rat model. Three different doses of hKGF-2 eye drops were administered topically and compared with two commercially available medicines as positive controls, including sodium hyaluronate (SH) eye drops and bovine basic fibroblast growth factor (bFGF) eye drops. The primary endpoints included tear secretion measurement, corneal fluorescein staining (CFS), tear film break-up time (TBUT), and histopathologic examination. For the safety pharmacology studies, the effects on the central nervous system (CNS) were determined using the functional observation battery (FOB) in rats. Additionally, the impacts on the cardiovascular and respiratory systems were analyzed in Beagle dogs by monitoring electrocardiogram (ECG), respiration (RESP), and blood pressure (BP).

**Results:**

Following 21 days of treatment, all topical hKGF-2 dosage groups (50, 100, 200 μg/mL) showed significant therapeutic effects, which were as effective as the bFGF and SH eye drops. The minimum effective dose (MED) of hKGF-2 in this study was 50 μg/mL. These therapeutic benefits included enhancing basal tear secretion and tear film stability, mitigating corneal damage, and promoting the recovery of corneal thickness as well as the restoration of conjunctival goblet cell counts. In safety pharmacology assessments, FOB results demonstrated that hKGF-2 treatment (20, 50, 100 μg per animal) had no adverse effects on the CNS of rats. Meanwhile, topical administration of hKGF-2 (160, 400, 800 μg per dog) did not elicit any adverse reactions in the cardiovascular or respiratory systems of beagles.

**Conclusion:**

Topical administration of hKGF-2 effectively ameliorated DED symptoms without adverse effects on the CNS, cardiovascular, and respiratory systems. These results indicated that hKGF-2 eye drops have potential as a safe and effective treatment for DED, and provided reliable preclinical evidence for its subsequent clinical trials and translational application.

## 1 Introduction

Dry eye disease (DED) is a common ocular surface disease that affects millions of people worldwide, impairing their vision and quality of life. The 2025 report of the Tear Film and Ocular Surface Society International Dry Eye Workshop III (TFOS DEWS III) defines dry eye as a multifactorial, symptomatic disease characterized by a loss of homeostasis of the tear film and/or ocular surface, in which tear film instability and hyperosmolarity, ocular surface inflammation and damage, and neurosensory abnormalities are etiological factors (Jones et al., 2025; Perez et al., 2025; Wolffsohn et al., 2025). It may collectively cause symptoms such as dryness, burning, photophobia, pain, and even visual disturbances.

With the increasing pervasiveness of digital screen use and global population aging, the prevalence of dry eye is likely to increase. However, the disease is often undertreated and underrecognized. Currently, artificial tears are the most widely used, but they can provide temporary relief from dry eye symptoms and have limited ability to solve problems (Clayton, 2018; Pflugfelder and de Paiva, 2017). Anti-inflammatory drugs, notably cyclosporine, face challenges including the slow onset of action and adverse effects such as ocular stinging and burning (Latkany, 2008; Lei et al., 2011). Long-term use of corticosteroid eye drops may also induce elevated intraocular pressure and cataracts (Clayton, 2018). Additionally, serum-based and growth factor medications such as bovine basic fibroblast growth factor (bFGF) eye drops, focusing on healing and repair, carry risks of corneal neovascularization and scar formation (Yan et al., 2013). Patients with moderate-to-severe DED usually require multiple medications, which compromises treatment adherence and increases the risk of drug-related adverse events. Thus, there is an urgent need to develop novel eye drop formulations that have excellent effects and minimal side effects.

Keratinocyte growth factor-2 (KGF-2), also known as fibroblast growth factor 10, is essential for the development, maintenance, and repair of ocular glands (Chen et al., 2014; Puk et al., 2009; Reneker et al., 2017). In our previous studies, hKGF-2 has been shown to markedly ameliorate corneal damage in rabbit models of alkali-induced and laser-induced corneal injury, via mechanisms including inflammation reduction, promotion of corneal epithelial stem cell regeneration and migration, and facilitation of corneal stromal fiber repair (Cai et al., 2019b; Wang et al., 2010). Additionally, KGF-2 has been found to increase tear meniscus height and area, heal corneal epithelium, reduce apoptosis of the corneal and conjunctival epithelial cells, and upregulate mucin secretion in acute inflammation-induced DED (Zheng et al., 2015). Ophthalmological studies have confirmed that KGF-2 ameliorates DED by alleviating ocular surface damage and suppressing inflammation through targeting the HMGB1/TLR4 pathway (Wang et al., 2025). However, its therapeutic effect in chronic DED remains poorly explored. In our previous preclinical studies, we completed toxicity evaluations of long-term topical administration of hKGF-2 eye drop (for the treatment of corneal damage) in rabbits (Cai et al., 2019a) and *Macaca fascicularis* (Li et al., 2021), as well as pharmacokinetic analyses in alkali-burned and intact rabbit eyes (Cai et al., 2015). Safety pharmacology evaluations are mandatory for novel drugs before clinical trials. However, to our knowledge, no such studies of hKGF-2 eye drops have been reported to date.

In the present study, we investigated the primary pharmacodynamics of hKGF-2 eye drops in two dry eye models: a BAC-induced rabbit model mimicking human evaporative DED and a SCOP-induced rat model mimicking human aqueous tear-deficient DED. Furthermore, this study is the first to systematically analyze the safety pharmacology of hKGF-2 eye drops. These preclinical data are critical for advancing hKGF-2 eye drops into clinical trials, and our findings support the potential of hKGF-2 as a therapeutic agent for the clinical treatment of DED.

## 2 Materials and methods

### 2.1 Reagents

hKGF-2 eye drops and their vehicle (detailed in Supplementary Table S1) were produced and provided by Wenzhou Medical University Biological and Natural Medicine Development Center Co., Ltd. (Wenzhou, China). Those eye drop formulations were stored at 4 °C, and were removed from the refrigerator and brought to room temperature before use. All eye drops meet sterile isotonic requirements. Sodium hyaluronate (SH) eye drops (Supplementary Table S1) were acquired by United Laboratories (Zhuhai, China). Bovine basic fibroblast growth factor (bFGF) eye drops (Supplementary Table S1) were supplied by Essex Bio-Technology (Zhuhai, China). Benzalkonium chloride (BAC, Cat# B6295) and sodium fluorescein (Cat# F6377) were purchased from Sigma-Aldrich (St. Louis, MO, United States). Scopolamine hydrobromide (SCOP, Cat# S817762) was purchased from Macklin (Shanghai, China). Hematoxylin and eosin staining kit (Cat# C0105M) and periodic acid-Schiff staining kit (Cat# C0142M) were obtained from Beyotime Biotechnology (Shanghai, China). Chlorpromazine hydrochloride injection (CPZ, Supplementary Table S1) was supplied by Harvest Pharmaceutical (Shanghai, China).

### 2.2 Animals

New Zealand rabbits (2.0–2.5 kg, 3–4 months old), Sprague-Dawley (SD) rats (SPF grade, 119–160 g, 4–5 weeks) and Beagle dogs (8.20–9.95 kg, 10–12 months old) were purchased from Pizhou Dongfang Breeding Co., Ltd. (Pizhou, China), Guangdong Weitonglihua Experimental Animal Technology Co., Ltd. (Guangdong, China) and Jiangsu Mas Biotechnology Co., Ltd. (Nantong, China), respectively. The animals were fed standard laboratory chow and drinking water freely. The ocular surfaces of all animals were assessed before grouping to exclude individuals with ocular abnormalities. The efficacy studies involving animals were approved by the Institutional Animal Care and Use Committee (IACUC) of Guangzhou General Pharmaceutical Research Institute (Guangzhou, China, Approval No. IA-PD2023013-01, IA-PD2023032-01). The safety pharmacology studies were approved by the IACUC of the Center for Drug Safety Evaluation and Research of Zhejiang University (Hangzhou, China, Approval No. IACUC-24-257, IACUC-24-301).

### 2.3 Primary pharmacodynamics of hKGF-2

#### 2.3.1 Primary pharmacodynamic in the BAC-induced rabbit dry eye model

**FIGURE 1:**
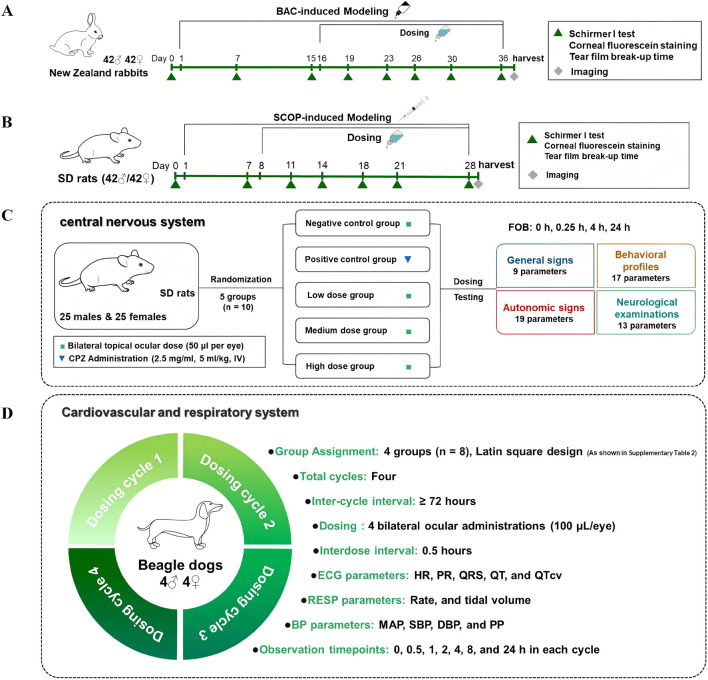
Experimental designs of efficacy and safety pharmacology studies **(A–D)**. The therapeutic effects were investigated in the BAC-induced rabbit **(A)** and SCOP-induced rat dry eye models **(B)**. Safety pharmacology studies on the central nervous system in rats **(C)** and on the cardiovascular and respiratory systems in beagles **(D)** were conducted.

0.1% BAC dissolved in phosphate-buffered saline (PBS) was used for the research. Seventy-two rabbits received bilateral topical instillation of 50 μL 0.1% BAC twice daily, whereas the normal control group received PBS instillation under the same regimen. After the 15-day induction, all rabbits except the normal control group were randomly allocated to the various therapeutic groups (n = 12 animals per group), consisting of three hKGF-2 eye drop groups (50, 100, 200 μg/mL), a bFGF eye drop group, and an SH eye drop group. Except for the SH group (administered 5 times a day with 50 μL per eye), other groups were given 100 μL of the respective eye drops or vehicle per eye four times daily. Those animals except the normal control group continued to receive bilateral BAC instillation throughout the entire 21-day treatment period. An interval of at least 2 h was maintained between consecutive instillations to avoid carryover effects of BAC and the therapeutic agents. Our study followed the well-established protocol for BAC-induced dry eye in rabbits (Xiong et al., 2008; Li et al., 2012) and the induction was maintained throughout the treatment period. Schirmer I test, tear film break-up time (TBUT), and corneal fluorescein staining (CFS) were performed at baseline (Day 0) and on Days 7, 15, 16, 19, 23, 26, 30, and 36. These time points were chosen to: (1) monitor the progressive development of dry eye during the 15-day BAC induction (Days 7, 15); (2) evaluate dynamic therapeutic responses during the treatment period (Days 16, 19, 23, 26, 30); and (3) assess sustained efficacy at the end of 21-day treatment (Day 36). Histopathological examinations were performed following euthanasia on day 37 (Figure 1A). Rabbits were anesthetized with sodium pentobarbital (30 mg/kg) via the marginal ear vein and euthanized with an overdose (150 mg/kg) via the same route.

#### 2.3.2 Primary pharmacodynamic in SCOP-induced rat dry eye model

SD rats (42 females and 42 males) were randomly divided into seven groups (12 rats per group), with groupings consistent with those in the rabbit experiment. The groups included a normal control group, three hKGF-2 eye drop groups (50, 100, 200 μg/mL), a bFGF eye drop group, and an SH eye drop group. SCOP was dissolved in normal saline to a final concentration of 12.5 mg/mL. Except for the normal control group, all rats received subcutaneous injection of 0.25 mL SCOP solution four times daily for 7 consecutive days. Thereafter, both the SCOP-induced modeling and topical administration with therapeutic agents or vehicle were maintained for 21 days. The dosing frequency was identical to that in the rabbit experiment, while the administered volume was reduced by half. Schirmer I test, TBUT, and CFS were performed at baseline (Day 0) and on Days 7, 11, 14, 18, 21, and 28 (Viau et al., 2008). This time course was designed to: (1) confirm the establishment of dry eye after 7 days of SCOP induction (Day 7); (2) detect early therapeutic effects during the initial phase of treatment (Day 11, 14); (3) evaluate peak efficacy during the model’s peak severity phase (Days 18, 21); and (4) confirm overall treatment efficacy at the end of the 21-day dosing regimen (Day 28). Final histopathological examinations were performed after euthanasia on day 29 (Figure 1B). Rats were anesthetized with sodium pentobarbital (45 mg/kg, i.p.) and euthanized with an overdose (150 mg/kg, i.p.).

#### 2.3.3 Schirmer I test

Tear volume was measured by the Schirmer test strip (Tianjin Jingming New Technology Development Co., Ltd., Tianjin, China) at the same time of the testing. Under anesthesia, the strip was placed in the inferior conjunctival sac of the eye by gently touching the eyelid and ocular surface, and it was left there for 5 min. The wet length of each strip was measured with calipers. Averages were based on measurements from both eyes.

#### 2.3.4 Corneal fluorescein staining (CFS) and tear film break-up time (TBUT)

Corneal epithelial damage was visualized via fluorescein staining of each eye. 1 μL of 1% sodium fluorescein solution (dissolved in saline) was introduced into the inferior conjunctival sac with a micropipette. Ninety seconds after three blinks, the test was performed under cobalt blue light using a slit lamp biomicroscope. Images of each eye were captured and graded. Each cornea was divided into four quadrants that were scored individually. The intensity of corneal fluorescein staining was rated on a 3-point scale: 0, no green staining; 1, slightly green punctate staining; 2, diffuse green punctate staining; 3, confluent fluorescein plaque/patch. The scores of the four areas were summed to obtain a final score, with a range of 0–12. For TBUT assessment, fluorescein staining was uniformly distributed over the corneal surface, and the TBUT was recorded from the final complete blink to the appearance of the first break point. Averages were based on measurements from both eyes.

#### 2.3.5 Hematoxylin-eosin (HE) staining and periodic acid-Schiff (PAS) staining

Corneal and conjunctival tissue sections were deparaffinized, rehydrated, and stained with hematoxylin and eosin staining in accordance with standard protocols and the instructions of the staining kit. Conjunctival sections were stained with periodic acid-Schiff staining following standard procedures. Three non-overlapping random fields of view were selected for counting, and the mean value was calculated.

### 2.4 Safety pharmacology studies of hKGF-2

#### 2.4.1 Safety pharmacology studies on the CNS in rats

Fifty SD rats (25 females and 25 males) were randomly divided into five groups (10 rats per group): a negative control group, a positive control group, and three graded-dose hKGF-2 eye drop groups (200, 500, 1,000 μg/mL). The chlorpromazine hydrochloride injection (CPZ, a central dopamine receptor antagonist, 25 mg/mL; as shown in Supplementary Table S1) was diluted to a concentration of 2.5 mg/mL with saline. Rats in the positive control group received this CPZ solution via tail vein intravenous injection at a dose of 5 mL/kg. Rats in the negative control group and the three hKGF-2 dose groups received bilateral ocular instillation of vehicle or the test formulations at a volume of 50 μL per eye, respectively (Figure 1C).

#### 2.4.2 Safety pharmacology studies on the cardiovascular and respiratory systems in beagle dogs

Eight qualified Beagle dogs (4 males and 4 females) were included in a four-period Latin square design study (grouping and experimental details are provided in Supplementary Table S2). The dogs were allocated into four groups: a negative control group (administered vehicle) and three hKGF-2 eye drop groups (200, 500, 1,000 μg/mL, respectively). All groups underwent a treatment period consisting of four administrations (bilateral instillations of approximately 100 μL per eye), with 30-min dosing intervals. This period was repeated for four cycles, with a minimum 72-h washout period between consecutive dosing cycles (Figure 1D).

#### 2.4.3 Functional observation battery (FOB)

FOB tests were performed at predose (0 h) and 0.25 h, 4, and 24 h post-dosing. As shown in Supplementary Table S3, A total of 58 parameters were evaluated in all animals. The tests were performed in an order starting with those having minimal impact on the animals, followed by those requiring more manual manipulation. To avoid bias, the FOB observer was blinded to animal and group information before the results came out.

#### 2.4.4 Electrocardiogram (ECG), respiration (RESP), and blood pressure (BP) monitoring

ECG, RESP, and BP were monitored using an EmkaPACK_4G non-invasive physiological telemetry system (Emka Technologies Inc., France) for conscious animals. Measurements were collected before dosing (0 h) and at 0.5, 1, 2, 4, 8, and 24 h after the final administration in each treatment cycle.

All Parameters are as follows:

- ECG parameters: heart rate (HR), PR Interval, QRS Interval, QT Interval, QTcv; QTcv was calculated using the following formula:

$$QTcv=QT-87\times \left(60/HR-1\right)$$

- RESP parameters: respiratory rate and tidal volume;
- BP parameters: mean arterial pressure (MAP), systolic blood pressure (SBP), diastolic blood pressure (DBP), and pulse pressure.

### 2.5 Statistical analysis

The data from our research were analyzed using GraphPad Prism 10.0 software (GraphPad Software, San Diego, CA, United States). Statistical analysis was performed using one-way ANOVA, and data are presented as the mean ± standard deviation (SD). A *P*-value of less than 0.05 was considered to indicate a statistically significant difference.

## 3 Results

### 3.1 hKGF-2 improved the tear secretion rate and tear film stability

**FIGURE 2:**
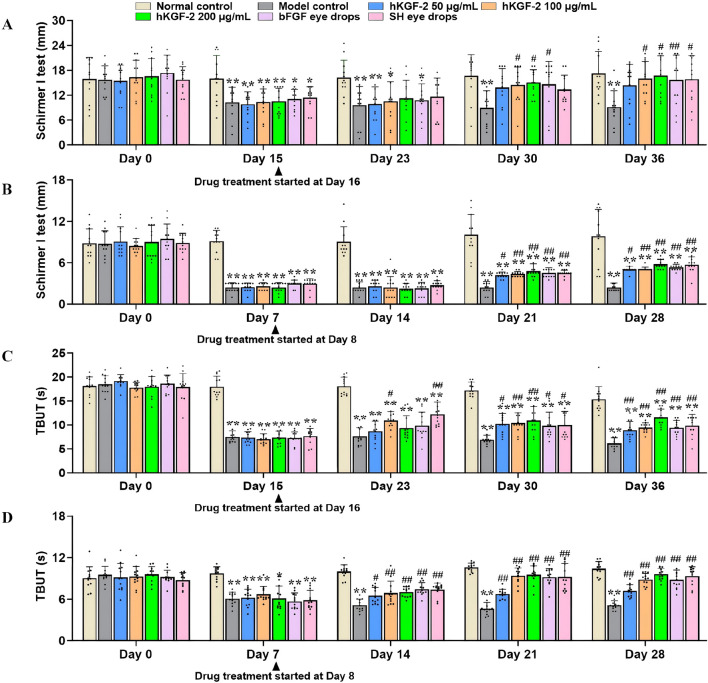
hKGF-2 treatment improves tear secretion rate and tear film stability **(A–D)**. Tear secretion rates of rabbits **(A)** and rats **(B)** were analyzed by the Schirmer I test. Tear film stability was detected by TBUT at the same time in rabbits **(C)** and rats **(D)**. The average of the results from both eyes is the data for one animal, and the data are shown as the mean ± SD (n = 12). ^*^ *P* < 0.05, ^**^ *P* < 0.01, and ^***^ *P* < 0.001, compared with normal control group. ^#^ *P* < 0.05, ^##^ *P* < 0.01, and ^###^ *P* < 0.001, compared with model control group.

To investigate whether hKGF-2 eye drops mitigate the tear film instability of DED, we established two animal models to mimic human DED. The key clinical endpoints, including Schirmer I test and TBUT, were observed on days 0, 7, 15, 19, 23, 26, 30, and 36 in rabbits (Figure 1A), and on days 0, 7, 11, 14, 18, 21, and 28 in rats (Figure 1B). As shown in Figures 2A,B, tear secretion and TBUT declined gradually over 15 days of BAC induction or 7 days of SCOP-induced modeling. However, tear secretion rate increased significantly after about 2 weeks of treatment in the medium- and high-dose hKGF-2 group compared with the model control group (*P <* 0.05), suggesting that hKGF-2 may alleviate DED by improving tear secretion. In addition, the TBUT levels were also higher in the hKGF-2-treated groups than in the model control group (Figures 2C,D). Notably, hKGF-2 demonstrated comparable therapeutic efficacy to two clinically approved DED therapeutics, bFGF and SH eye drops.

### 3.2 hKGF-2 improved corneal epithelial integrity and regularity

**FIGURE 3:**
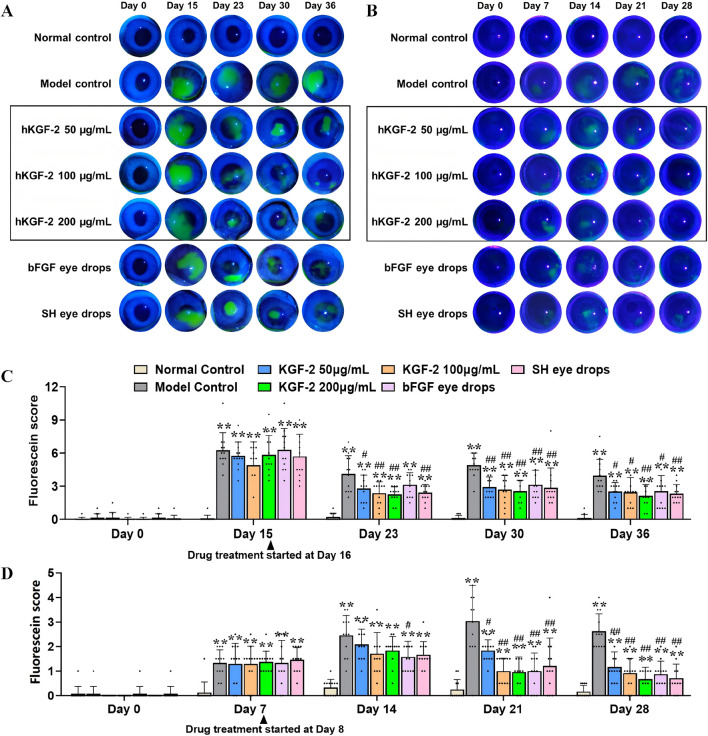
hKGF-2 improved corneal and conjunctival damage caused by DED **(A–D)**. Corneal epithelial damage was stained with fluorescein sodium in rabbits **(A)** and rats **(B)**. Fluorescein scores were analyzed in rabbits **(C)** and rats **(D)**. The average of the results from both eyes is the data for one animal, and the data are shown as the mean ± SD (n = 12). ^*^ *P* < 0.05, ^**^ *P* < 0.01, and ^***^ *P* < 0.001, compared with normal control group. ^#^ *P* < 0.05, ^##^ *P* < 0.01, and ^###^ *P* < 0.001, compared with model control group.

Corneal epithelial cells are significantly impaired once tear film instability has occurred. To evaluate the protective effects of hKGF-2 on the ocular surface, CFS was performed using a slit lamp for all groups of rabbits and rats (Figure 3). The results demonstrated that the fluorescein staining scores were markedly elevated in model animals relative to the normal control group, whereas hKGF-2 significantly reduced these scores after 8 days of administration in the rabbit model and 11 days in the rat model. By contrast, hKGF-2 eye drops produced a comparable therapeutic effect to the other two drugs. These findings suggest that hKGF-2 had reparative effects on the corneas of dry eye animals.

### 3.3 hKGF-2 ameliorated histopathological alterations caused by DED

**FIGURE 4:**
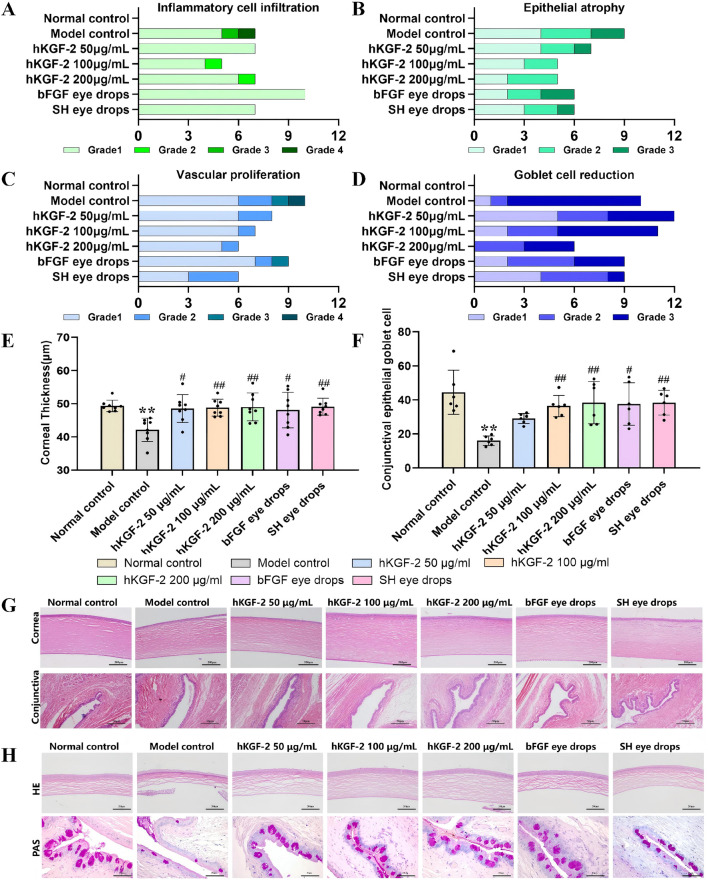
hKGF-2 ameliorated histopathological alterations caused by DED **(A–D)**. Inflammatory cell infiltration **(A)**, epithelial atrophy **(B)**, and vascular proliferation **(C)** in the rabbit corneas were analyzed based on the HE staining (n = 12). Conjunctival Goblet cell reduction of rabbits **(D)** was evaluated based on the HE staining (n = 12). Corneal thickness **(E)**, n = 8) and conjunctival goblet cell counts **(F)**, n = 6) of rats were normalized. Representative images of hematoxylin and eosin (HE) staining of the rabbit cornea and conjunctiva are shown **(G)**. HE staining of the cornea and PAS staining of the conjunctiva in rats were obtained **(H)**. Data are shown as the mean ± SD. Grades: 1 = Minimal; 2 = Mild; 3 = Moderate; 4 = Severe; 5 = Critical. ^*^ *P* < 0.05, ^**^ *P* < 0.01, and ^***^ *P* < 0.001, compared with normal control group. ^#^ *P* < 0.05, ^##^ *P* < 0.01, and ^###^ *P* < 0.001, compared with model control group.

In the rabbit model, 0.1% BAC was topically administered to recapitulate human evaporative DED, which resulted in marked corneal epithelial atrophy, disordered cellular arrangement, neovascular proliferation, and inflammatory cell infiltration (Figure 4G). A reduction in conjunctival goblet cells was also observed after modeling. The severity and incidence of histopathological alterations were shown in Figures 4A–D. Following 21 days of treatment, all hKGF-2 groups showed improvements in corneal epithelial atrophy, corneal inflammatory cell infiltration, and conjunctival goblet cell reduction compared with the model control group. Furthermore, the medium- and high-dose hKGF-2 groups demonstrated similar efficacy in promoting corneal wound repair relative to both the bFGF and SH eye drop groups.

In the SCOP-induced rat model, the model control group showed a statistically significant reduction in corneal thickness (*P* < 0.01), without other obvious abnormalities (Figure 4H). Additionally, the model control group showed a significant decrease in conjunctival goblet cells relative to the normal control group (*P* < 0.01). Following 21 days of continuous administration, all hKGF-2 groups significantly restored corneal thickness (Figure 4E). While low-dose KGF-2 only induced a marginal, non-significant rise in goblet cell numbers (*P* > 0.05), both the medium- and high-dose groups significantly increased the conjunctival goblet cell numbers (*P* < 0.01, Figure 4F).

### 3.4 Topical application of hKGF-2 exerted no adverse effects on the CNS of rats

To elucidate the impacts on the CNS, we conducted this preclinical study in rats to support subsequent clinical trial initiation. CPZ is a classic central dopamine receptor antagonist that exerts potent inhibitory effects on the CNS (Leucht et al., 2024). The positive control group was administered CPZ (0.5 mg/kg, IV), whereas other rats were given hKGF-2 (20, 50, and 100 μg per animal, respectively) or vehicle, following the FOB test (see Supplementary Table S3). These 58 FOB parameters were used to evaluate CNS-related effects in rats across four key domains: general signs, behavioral profiles, neurological examinations, and autonomic signs.

**FIGURE 5:**
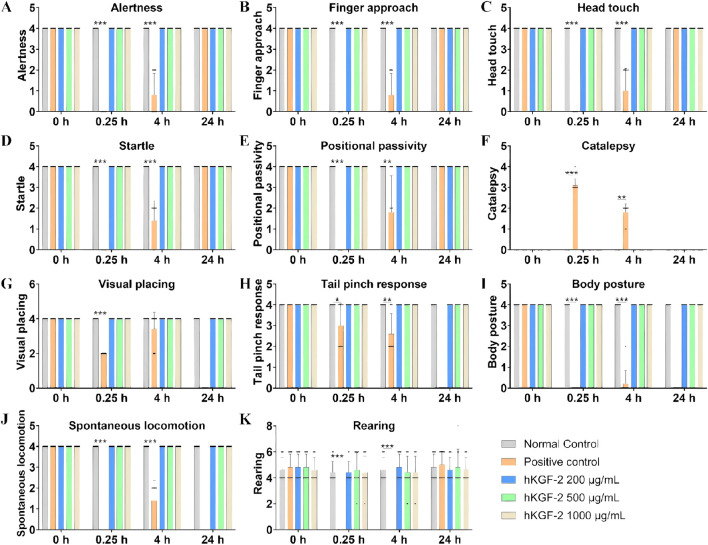
Effects of hKGF-2 on the CNS of rats: behavioral profiles **(A–K)**. Behavioral profiles, including alertness **(A)**, finger approach **(B)**, head touch **(C)**, startle **(D)**, positional passivity **(E)**, visual placing **(G)**, tail pinch response **(H)**, body posture **(I)**, spontaneous locomotion **(J)**, and rearing **(K)**, were recorded and scored (score range for each parameter: 0 to 8; reference standard: 4). Catalepsy **(F)** were scored and analyzed (score range for each parameter: 0 to 4; reference standard: 0). Data are shown as the mean ± SD (n = 10). Compared with the negative control group, ^*^ *P* < 0.05, ^**^ *P* < 0.01, and ^***^ *P* < 0.001.

**FIGURE 6:**
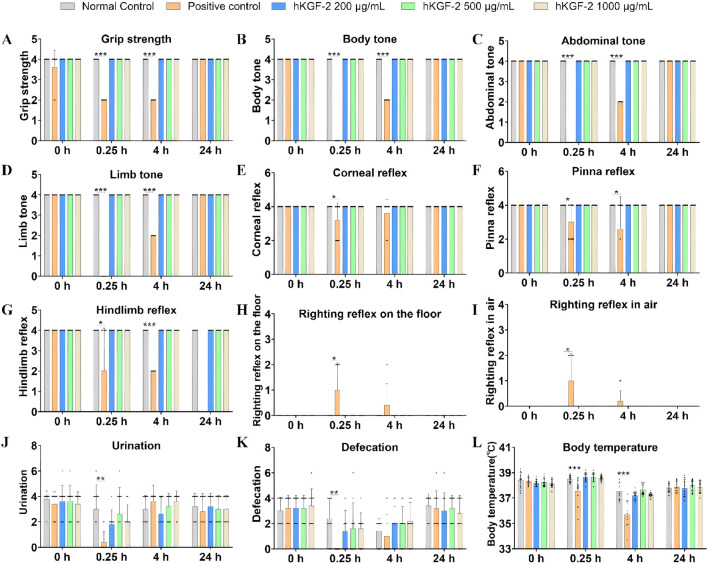
Effects of hKGF-2 on the CNS of rats: neurological examinations and autonomic signs **(A–L)**. Neurological examinations, including grip strength **(A)**, body tone **(B)**, abdominal tone **(C)**, limb tone **(D)**, corneal reflex **(E)**, pinna reflex **(F)**, and hindlimb reflex **(G)**, were recorded and scored (score range for each parameter: 0 to 8; reference standard: 4). Righting reflex on the floor **(H)**, righting reflex in air **(I)** were scored and analyzed (score range for each parameter: 0 to 4; reference standard: 0). Autonomic signs, including urination **(J)**, defecation **(K)**, and body temperature, were recorded and analyzed. Data are shown as the mean ± SD (n = 10). Compared with the negative control group, ^*^ *P* < 0.05, ^**^ *P* < 0.01, and ^***^ *P* < 0.001.

For general signs, cage-side observations revealed no statistically significant abnormalities in any hKGF-2 dose group or the positive control group relative to the negative control group (*P* > 0.05; Supplementary Table S5). Rats in the positive control group (CPZ-treated) exhibited extensive CNS depression-related abnormalities, including impaired behavioral responses, compromised neurological reflexes, disrupted autonomic functions, hypothermia, and so on (see Figures 5, 6; Supplementary Tables S6, S7). Apart from the above symptoms, no other abnormalities were noted in the remaining parameters (Supplementary Tables S8, S9). In summary, we observed FOB’s 58 parameters on four CNS-related domains, including general signs, behavioral profiles, neurological examinations, and autonomic signs of rats. No significant abnormalities of the CNS (*P* > 0.05) were observed in any of the three hKGF-2 groups relative to the negative control group.

### 3.5 Topical application of hKGF-2 had no adverse effects on the cardiovascular and respiratory systems in beagle dogs

In accordance with relevant technical guidelines, Beagle dogs were administered hKGF-2 (160, 400, and 800 μg per animal, respectively) or vehicle to observe the effects on the cardiovascular and respiratory systems and identify potential unexpected pharmacological effects of this novel drug. General clinical observations revealed no abnormalities across all treatment groups.

**FIGURE 7:**
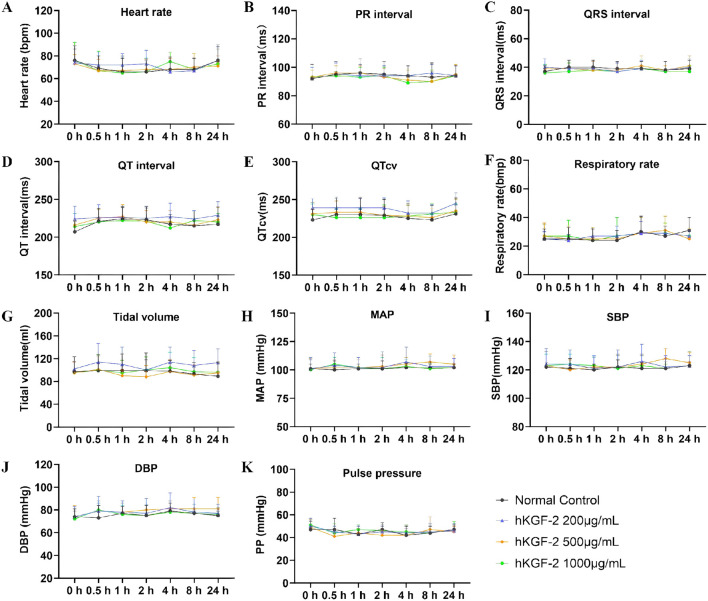
Effects of different doses of hKGF-2 on electrocardiogram, respiration, and blood pressure in beagles **(A–K)**. ECG parameters were measured, including heart rate **(A)**, PR interval **(B)**, QRS interval **(C)**, QT interval **(D)**, and QTcv **(E)**. Parameters of the respiratory system were measured, including respiratory rate **(F)** and tidal volume **(G)**. BP parameters were monitored, including mean arterial pressure **(H)**, systolic blood pressure **(I)**, diastolic blood pressure **(J)**, and pulse pressure **(K)**. Data are shown as the mean ± SD (n = 8).

ECG monitoring showed that none of the three hKGF-2 groups showed any significant differences (*P* > 0.05) relative to the negative control group in heart rate, PR interval, QRS interval, QT interval, or QTcv (*P* > 0.05; Figures 7A–E). Furthermore, monitoring of respiratory parameters (tidal volume and respiratory rate) revealed no obvious difference at any time point (*P* > 0.05; Figures 7F,G). Similarly, the results from blood pressure monitoring (including MAP, SBP, DBP, and PP) indicated no adverse impacts of the hKGF-2 eye drops (Figures 7H–K).

## 4 Discussion

The prevalence of DED is rising steadily, thereby posing significant challenges for patients and the healthcare system worldwide. Clinically, DED mainly includes two types: over-evaporative and aqueous tear-deficient DED (Pflugfelder and de Paiva, 2017; Rahman et al., 2021). Given the multifactorial pathogenesis of DED, a single animal model cannot fully recapitulate its complex clinical phenotypes. Therefore, we utilized two distinct animal models to investigate the efficacy of hKGF-2 eye drops. BAC-induced over-evaporative DED in rabbits and SCOP-induced aqueous tear-deficient DED in rats were successfully established. By comparing the various dose groups with the model and positive control groups, the studies preliminarily identified the effective dose of hKGF-2 eye drops in these animal models.

Tear secretion volume, TBUT, and CFS are core diagnostic metrics for DED (Rolando and Merayo-Lloves, 2022; Wolffsohn et al., 2025). Compared to the model control groups, significant differences were observed in those metrics after 2–3 weeks of treatment, suggesting that hKGF-2 ameliorates dry eye by improving tear secretion rate, stabilizing the tear film, and preserving corneal epithelial integrity. The therapeutic effect of the medium- or high-dose group is similar to the positive control group. hKGF-2 exhibits superior efficacy to bFGF in promoting reepithelialization, accelerating cell migration, and attenuating scar formation in a corneal alkali burn model (Cai et al., 2019b). We hypothesize that this therapeutic advantage extends to dry eye syndrome treatment, and will conduct in-depth investigations into the underlying mechanism in future studies. In addition, histopathological analyses in all hKGF-2 groups confirmed the alleviation of corneal pathological lesions and the restoration of conjunctival goblet cell count. Collectively, hKGF-2 eye drops significantly improved DED in both BAC-induced rabbit and scopolamine-induced rat models, and the minimum effective dose (MED) in this study was 50 μg/mL. Clinical epidemiological studies demonstrated a female predominance in DED due to hormonal disparities (Perez et al., 2025; Sriprasert et al., 2015), yet no significant sex-related differences were observed in our animal studies. Currently, this hKGF-2 eye drop has been approved by the Center for Drug Evaluation of China to initiate clinical trials. Given the inherent differences between animal models and human subjects, this sex-related disparity will be a key focus in subsequent clinical trials. In summary, these results provide a scientific basis for the formulation of clinical dosing regimens for hKGF-2 eye drops.

Safety pharmacology studies are an essential prerequisite for the clinical translation of novel pharmaceuticals. To ensure the rigor and reproducibility of the experimental procedures, our studies complied with the ICH S7A guideline ICHS7A, 2000 and S7B guideline ICHS7B, 2005. All safety pharmacology studies were conducted in adherence to Good Laboratory Practice principles. The rodent central nervous system, and conscious non-rodent telemetry cardiovascular and respiratory function were employed. Doses of 200, 500, and 1,000 μg/mL were selected as the general pharmacological dosages, which were markedly higher than MED in our efficacy studies. In rats, the FOB was conducted, totaling 58 parameters across the domains of general signs, behavioral profiles, neurological examinations, and autonomic signs. The results demonstrate that single ocular administration (20, 50, 100 μg/eye) exerted no effects on the central nervous system.

In beagles, dogs received the hKGF-2 via ocular instillation at doses of 0, 160, 400, and 800 μg in each dosing cycle. It found that hKGF-2 exerted no significant effects on the cardiovascular or respiratory systems, by ECG, RESP, and BP monitoring. These findings suggest that hKGF-2 eye drops exhibited good therapeutic efficacy and safety, which provided critical translational preclinical data.

## 5 Conclusion

hKGF-2 eye drops enhanced tear secretion rate, tear film stability, and corneal epithelial integrity and regularity. They can also ameliorate histopathological alterations caused by dry eye. Furthermore, topical application of hKGF-2 exerted no adverse effects on the CNS in rats or on the cardiovascular and respiratory system in beagles. Dry eye disease has unmet clinical needs. Our results demonstrated that hKGF-2 eye drops are a promising therapeutic candidate for the management of DED, and provided key translational data.

## Data Availability

The original contributions presented in the study are included in the article/Supplementary Material, further inquiries can be directed to the corresponding authors.

## References

[B1] Cai J. Dou G. Zheng L. Yang T. Jia X. Tang L. (2015). Pharmacokinetics of topically applied recombinant human keratinocyte growth factor-2 in alkali-burned and intact rabbit eye. Exp. Eye Res. 136, 93–99. 10.1016/j.exer.2015.05.006 25987499

[B2] Cai J. Li W. Li J. Zhou Q. Huang Y. Li X. (2019a). Toxicology study of long-term administration of rhKGF-2 eye drops on rabbit corneas. Regul. Toxicol. Pharmacol. 103, 189–195. 10.1016/j.yrtph.2019.02.002 30735698

[B3] Cai J. Zhou Q. Wang Z. Guo R. Yang R. Yang X. (2019b). Comparative analysis of KGF-2 and bFGF in prevention of excessive wound healing and scar formation in a corneal alkali burn model. Cornea 38 (11), 1430–1437. 10.1097/ICO.0000000000002134 31490279

[B4] Chen Z. Huang J. Liu Y. Dattilo L. K. Huh S.-H. Ornitz D. (2014). FGF signaling activates a Sox9-Sox10 pathway for the formation and branching morphogenesis of mouse ocular glands. Development 141 (13), 2691–2701. 10.1242/dev.108944 24924191 PMC4067963

[B5] Clayton J. A. (2018). Dry eye. N. Engl. J. Med. 378 (23), 2212–2223. 10.1056/NEJMra1407936 29874529

[B6] Jones L. Craig J. P. Markoulli M. Karpecki P. Akpek E. K. Basu S. (2025). TFOS DEWS III: Management and therapy. Am. J. PsychiatryInvestigative Ophthalmol. and Vis. Sci. 279, 289–386. 10.1016/j.ajo.2025.05.039 40467022

[B7] Latkany R. (2008). Dry eyes: etiology and management. Curr. Opin. Ophthalmol. 19 (4), 287–291. 10.1097/ICU.0b013e3283023d4c 18545008

[B8] Lei H. L. Ku W. C. Sun M. H. Chen K. J. Lai J. Y. Sun C. C. (2011). Cyclosporine a eye drop-induced elongated eyelashes: a case report. Case Rep. Ophthalmol. 2 (3), 398–400. 10.1159/000335281 22291642 PMC3268523

[B9] Leucht S. Priller J. Davis J. M. (2024). Antipsychotic drugs: a concise review of history, classification, indications, mechanism, efficacy, side effects, dosing, and clinical application. Am. J. Psychiatry 181 (10), 865–878. 10.1176/appi.ajp.20240738 39350614

[B10] Li C. Song Y. Luan S. Wan P. Li N. Tang J. (2012). Research on the stability of a rabbit dry eye model induced by topical application of the preservative benzalkonium chloride. PLoS One 7 (3), e33688. 10.1371/journal.pone.0033688 22438984 PMC3306287

[B11] Li L. Li L. Chen Q. Yang X. Hui Q. Al-Azzani H. (2021). Toxicity evaluation of long-term topical application of recombinant human keratinocyte growth factor-2 eye drops on *Macaca fascicularis* . Front. Pharmacol. 12, 740726. 10.3389/fphar.2021.740726 34621172 PMC8490875

[B12] Perez V. L. Chen W. Craig J. P. Dogru M. Jones L. Stapleton F. (2025). TFOS DEWS III: executive summary. Am. J. Ophthalmol. S0002-9394, 00514–00518. 10.1016/j.ajo.2025.09.035 41005521

[B13] Pflugfelder S. C. de Paiva C. S. (2017). The pathophysiology of dry eye disease: what we know and future directions for research. Ophthalmology 124 (11), S4–S13. 10.1016/j.ophtha.2017.07.010 29055361 PMC5657523

[B14] Puk O. Esposito I. So¨ker T. Lo¨ster J. Budde B. Nu¨rnberg P. (2009). A new Fgf10 mutation in the mouse leads to atrophy of the Harderian gland and slit-eye phenotype in heterozygotes: a novel model for dry-eye disease? Investig. Ophthalmol. Vis. Sci. 50 (9), 4311–4318. 10.1167/iovs.09-3451 19407009

[B15] Rahman M. M. Kim D. H. Park C.-K. Kim Y. H. (2021). Experimental models, induction protocols, and measured parameters in dry eye disease: focusing on practical implications for experimental research. Int. J. Mol. Sci. 22 (22), 12102. 10.3390/ijms222212102 34830010 PMC8622350

[B16] Reneker L. W. Wang L. Irlmeier R. T. Huang A. J. W. (2017). Fibroblast growth factor receptor 2 (FGFR2) is required for Meibomian gland homeostasis in the adult mouse. Investig. Ophthalmol. Vis. Sci. 58 (5), 2638–2646. 10.1167/iovs.16-21204 28510629 PMC5444547

[B17] Rolando M. Merayo-Lloves J. (2022). Management strategies for evaporative dry eye disease and future perspective. Curr. Eye Res. 47 (6), 813–823. 10.1080/02713683.2022.2039205 35521685

[B18] Sriprasert I. Warren D. W. Mircheff A. K. Stanczyk F. Z. (2015). Dry eye in postmenopausal women: a hormonal disorder. Menopause 23 (3), 343–351. 10.1097/GME.0000000000000530 26529614

[B19] Viau S. Maire M. A. Pasquis B. Grégoire S. Fourgeux C. Acar N. (2008). Time course of ocular surface and lacrimal gland changes in a new scopolamine-induced dry eye model. Graefes Arch. Clin. Exp. Ophthalmol. 246 (6), 857–867. 10.1007/s00417-008-0784-9 18357464

[B20] Wang X. Zhou X. Ma J. Tian H. Jiao Y. Zhang R. (2010). Effects of keratinocyte growth factor-2 on corneal epithelial wound healing in a rabbit model of carbon dioxide laser injury. Biol. Pharm. Bull. 33 (6), 971–976. 10.1248/bpb.33.971 20522961

[B21] Wang Y. Xu Z. Wei L. Lu Y. Shi Y. Wen S. (2025). KGF-2 alleviates dry eye disease by regulating the HMGB1/TLR4 pathway. Investig. Ophthalmol. Vis. Sci. 66 (4), 28. 10.1167/iovs.66.4.28 40227178 PMC12007672

[B22] Wolffsohn J. S. Benítez-Del-Castillo J. M. Loya-Garcia D. Inomata T. Iyer G. Liang L. (2025). TFOS DEWS III: diagnostic methodology. Am. J. Ophthalmol. 279, 387–450. 10.1016/j.ajo.2025.05.033 40451408

[B23] Xiong C. Chen D. Liu J. Liu B. Li N. Zhou Y. (2008). A rabbit dry eye model induced by topical medication of a preservative benzalkonium chloride. Investig. Ophthalmol. Vis. Sci. 49 (5), 1850–1856. 10.1167/iovs.07-0720 18436819

[B24] Yan L. Wu W. Wang Z. Li C. Lu X. Duan H. (2013). Comparative study of the effects of recombinant human epidermal growth factor and basic fibroblast growth factor on corneal epithelial wound healing and neovascularization *in vivo* and *in vitro* . Ophthalmic Res. 49 (3), 150–160. 10.1159/000343775 23258255

[B25] Zheng W. Ma M. Du E. Zhang Z. Jiang K. Gu Q. (2015). Therapeutic efficacy of fibroblast growth factor 10 in a rabbit model of dry eye. Mol. Med. Rep. 12 (5), 7344–7350. 10.3892/mmr.2015.4368 26459017 PMC4626165

