# Supplementary material for: Efficacy and safety of topical human keratinocyte growth factor-2 for dry eye disease: evidence from in vivo studies
Source: Front Pharmacol. 2026 Mar 25;17:1800966. doi: 10.3389/fphar.2026.1800966 (PMC13057480; doi:10.3389/fphar.2026.1800966)
Supplement: Supplementary file 1 [file Table1.docx]

Supplementary Material

**Supplementary Table 1** Reagents and pharmaceuticals employed in the study

| Experiment | Name | Concentration of active substance | Specifications | Batch numbers | Manufacturers |
| --- | --- | --- | --- | --- | --- |
| Efficacy evaluation | hKGF-2 eye drops vehicle | None | 1 ml/tube | 20230601  20231005 | Wenzhou Medical University Biological and Natural Medicine Development Center Co., Ltd (Wenzhou, China) |
|  | hKGF-2 eye drops | 50 μg/ml | 1 ml/tube | 20230602  20231006 |  |
|  |  | 100 μg/ml | 1 ml/tube | 20230603  20231007 |  |
|  |  | 200 μg/ml | 1 ml/tube | 20230604  20231008 |  |
|  | bFGF eye drops | 4200 IU/ml | 5 ml/tube | 02230211  02230202  02230209 | Zhuhai Essex Bio-Pharmaceutical Co., Ltd. (Zhuhai, China) |
|  | SH eye drops | 1 mg/ml | 5 ml/tube | 29076104  35076103 | Zhuhai United Laboratories Co., Ltd. (Zhuhai, China) |
| Safety Pharmacology studies | hKGF-2 eye drops vehicle | None | 5 ml/tube | 20240304 | Wenzhou Medical University Biological and Natural Medicine Development Center Co., Ltd (Wenzhou, China) |
|  | hKGF-2 eye drops | 200 μg/ml | 5 ml/tube | 20240305 |  |
|  |  | 500 μg/ml | 5 ml/tube | 20240406 |  |
|  |  | 1000 μg/ml | 5 ml/tube | 20240407 |  |
|  | CPZ injection | 25 mg/ml | 2 ml/tube | 37220901 | Shanghai Harvest Pharmaceutical Co., Ltd. (Shanghai, China) |

**Supplementary Table 2** Animals and the dosage of safety pharmacology in beagle dogs

| Cage number | Gender | Treatment cycle and animal ID | | | |
| --- | --- | --- | --- | --- | --- |
|  |  | Ⅰ | Ⅱ | Ⅲ | Ⅳ |
| 1 | Male | 4M01 | 1M02 | 2M03 | 3M04 |
| 2 | Male | 1M01 | 2M02 | 3M03 | 4M04 |
| 3 | Male | 3M01 | 4M02 | 1M03 | 2M04 |
| 4 | Male | 2M01 | 3M02 | 4M03 | 1M04 |
| 5 | Female | 1F01 | 2F02 | 3F03 | 4F04 |
| 6 | Female | 4F01 | 1F02 | 2F03 | 3F04 |
| 7 | Female | 2F01 | 3F02 | 4F03 | 1F04 |
| 8 | Female | 3F01 | 4F02 | 1F03 | 2F04 |

●1M01–1M04 and 1F01–1F04 belong to the negative control group; 2M01–2M04 and 2F01–2F04, 3M01–3M04 and 3F01–3F04, 4M01–4M04 and 4F01–4F04 belong to the low, medium, and high dose groups of hKGF-2 eye drops, with concentrations of 200, 500, and 1000 μg/ml, respectively.

**Supplementary Table 3** Operation sequence and parameters of FOB

| **Operation sequence** | **Parameters** | **Score range** | **Reference** | **Operation sequence** | **Parameters** | **Score range** | **Reference** |
| --- | --- | --- | --- | --- | --- | --- | --- |
| **1** | Food consumption | 0/1 | NA | **30** | Startle response | 0–8 | 4 |
| **2** | Water consumption | 0/1 | NA | **31** | Heart rate | 0–4 | 0 |
| **3** | Animal sleeping | 0/1 | NA | **32** | Positional passivity | 0–8 | 4 |
| **4** | Behavioral inactivity | 0/1 | NA | **33** | Catalepsy | 0–4 | 0 |
| **5** | Locomotion | 0/1 | NA | **34** | Visual placing | 0–8 | 4 |
| **6** | Piloerection  (in cage) | 0/1 | NA | **35** | Grip strength | 0–8 | 4 |
| **7** | Attacking cage mates | 0/1 | NA | **36** | Corneal reflex | 0–8 | 4 |
| **8** | Vocalization  (in cage) | 0/1 | NA | **37** | Pinna reflex | 0–8 | 4 |
| **9** | Grooming behavior  (in cage) | 0/1 | NA | **38** | Body tone | 0–8 | 4 |
| **10** | Alertness | 0–8 | 4 | **39** | Abdominal tone | 0–8 | 4 |
| **11** | Finger approach | 0–8 | 4 | **40** | Limb tone | 0–8 | 4 |
| **12** | Head touch | 0–8 | 4 | **41** | Hindlimb reflex | 0–8 | 4 |
| **13** | Startle | 0–8 | 4 | **42** | Skin color | 0–8 | 4 |
| **14** | Body posture | 0–8 | 4 | **43** | Cyanosis | 0–4 | 0 |
| **15** | Spontaneous locomotion | 0–8 | 4 | **44** | Palpebral closure | 0–4 | 0 |
| **16** | Ataxic gait | 0–4 | 0 | **45** | Exophthalmos | 0–4 | 0 |
| **17** | Hypotonic gait | 0–4 | 0 | **46** | Pupil size | 0–8 | 4 |
| **18** | Convulsion | 0–4 | 0 | **47** | Lacrimation | 0–8 | 4 |
| **19** | Seizure | 0–4 | 0 | **48** | Chromodacryorrhea | 0–4 | 0 |
| **20** | Writhing | 0–4 | 0 | **49** | Salivation | 0–8 | 4 |
| **21** | Abnormal behavior | 0–4 | 0 | **50** | Tail pinch sensitivity | 0–8 | 4 |
| **22** | Tail position | 0–4 | 0 | **51** | Righting reflex on the floor | 0–4 | 0 |
| **23** | Piloerection | 0–4 | 0 | **52** | Righting reflex in air | 0–4 | 0 |
| **24** | Grooming Behavior | 0–4 | 0 | **53** | Aggression or irritability | 0–4 | 0 |
| **25** | Rearing | 0–8 | 4 | **54** | Abnormal vocalization | 0–4 | 0 |
| **26** | Urination | 0–8 | 4 | **55** | Consistency of feces | 0–8 | 4 |
| **27** | Defecation | 0–8 | 4 | **56** | Urine color | 0–4 | 0 |
| **28** | Respiration | 0–8 | 4 | **57** | Death | 0/1 | 0 |
| **29** | Tremor | 0–4 | 0 | **58** | Body temperature | NA | NA |

●The scoring system is defined as follows:

**a**. For parameters that could be below normal is possible. The reference value is set at 4. Scores range from 0 to 4 for values below the reference, and from 4 to 8 for values above the reference.

**b**. For parameters that are only above reference: The reference value is set at 0. Scores range from 0 to 4 for values above the reference.

**c**. For non-gradable findings (e.g., mortality): A binary score is used: 0 (absent) and 1 (present).

**d**. Body temperature: This parameter is not scored; the actual measured value is recorded.

**e.** NA: not available

**Supplementary Table 4** Number and severity of corneal and conjunctival lesions

| Histopathological examination | | | | Normal control | Model control | hKGF-2  50 μg/ml | hKGF-2  100 μg/ml | hKGF-2  200 μg/ml | bFGF eye drops | SH eye drops |
| --- | --- | --- | --- | --- | --- | --- | --- | --- | --- | --- |
| **cornea** | Totality | | | 12 | 12 | 12 | 12 | 12 | 12 | 12 |
|  | Inflammatory cell infiltration | Grades | 1 | 0 | 5 | 7 | 4 | 6 | 10 | 7 |
|  |  |  | 2 | 0 | 0 | 0 | 1 | 1 | 0 | 0 |
|  |  |  | 3 | 0 | 1 | 0 | 0 | 0 | 0 | 0 |
|  |  |  | 4 | 0 | 1 | 0 | 0 | 0 | 0 | 0 |
|  |  | Total incidence | | 0 | 7 | 7 | 5 | 7 | 10 | 7 |
|  | Epithelial atrophy | Grades | 1 | 0 | 4 | 4 | 3 | 2 | 2 | 3 |
|  |  |  | 2 | 0 | 3 | 2 | 2 | 3 | 2 | 2 |
|  |  |  | 3 | 0 | 2 | 1 | 0 | 0 | 2 | 1 |
|  |  | Total incidence | | 0 | 9 | 7 | 5 | 5 | 6 | 6 |
|  | Vascular proliferation | Grades | 1 | 0 | 6 | 6 | 6 | 5 | 7 | 3 |
|  |  |  | 2 | 0 | 2 | 2 | 1 | 1 | 1 | 3 |
|  |  |  | 3 | 0 | 1 | 0 | 0 | 0 | 1 | 0 |
|  |  |  | 4 | 0 | 1 | 0 | 0 | 0 | 0 | 0 |
|  |  | Total incidence | | 0 | 10 | 8 | 7 | 6 | 9 | 6 |
| **Conjunctiva** | Totality | | | 12 | 12 | 12 | 12 | 12 | 12 | 12 |
|  | Goblet cell reduction | Grades | 1 | 0 | 1 | 5 | 2 | 0 | 2 | 4 |
|  |  |  | 2 | 0 | 1 | 3 | 3 | 3 | 4 | 4 |
|  |  |  | 3 | 0 | 8 | 4 | 6 | 3 | 3 | 1 |
|  |  | Total incidence | | 0 | 10 | 12 | 11 | 6 | 9 | 9 |

● Grades: 1 = Minimal; 2 = Mild; 3 = Moderate; 4 = Severe; 5 = Critical.

**Supplementary Table 5** Effects of hKGF-2 on the rats' CNS: general signs

| Timepoints | Groups | Parameters of general signs | | | | | | | | |
| --- | --- | --- | --- | --- | --- | --- | --- | --- | --- | --- |
|  |  | Food Consumption | Water Consumption | Animal Sleeping | Behavioral Inactivity | Locomotion | Piloerection  (in cage) | Attacking Cage Mates | Vocalization  (in cage) | Grooming Behavior  (in cage) |
| 0 h  (predose) | NC | 0/10 | 0/10 | 0/10 | 10/10 | 0/10 | 0/10 | 0/10 | 0/10 | 0/10 |
|  | PC | 0/10 | 0/10 | 0/10 | 9/10 | 1/10 | 0/10 | 0/10 | 0/10 | 0/10 |
|  | LD | 0/10 | 0/10 | 0/10 | 9/10 | 1/10 | 0/10 | 0/10 | 0/10 | 0/10 |
|  | MD | 0/10 | 0/10 | 0/10 | 10/10 | 0/10 | 0/10 | 0/10 | 0/10 | 0/10 |
|  | HD | 0/10 | 0/10 | 0/10 | 10/10 | 0/10 | 0/10 | 0/10 | 0/10 | 0/10 |
| 0.25 h | NC | 0/10 | 0/10 | 0/10 | 9/10 | 1/10 | 0/10 | 0/10 | 0/10 | 0/10 |
|  | PC | 0/10 | 0/10 | 0/10 | 10/10 | 0/10 | 0/10 | 0/10 | 0/10 | 0/10 |
|  | LD | 0/10 | 0/10 | 0/10 | 10/10 | 0/10 | 0/10 | 0/10 | 0/10 | 0/10 |
|  | MD | 0/10 | 0/10 | 0/10 | 10/10 | 0/10 | 0/10 | 0/10 | 0/10 | 0/10 |
|  | HD | 0/10 | 0/10 | 0/10 | 10/10 | 0/10 | 0/10 | 0/10 | 0/10 | 0/10 |
| 4 h | NC | 0/10 | 0/10 | 0/10 | 9/10 | 1/10 | 0/10 | 0/10 | 0/10 | 0/10 |
|  | PC | 0/10 | 0/10 | 0/10 | 10/10 | 0/10 | 0/10 | 0/10 | 0/10 | 0/10 |
|  | LD | 0/10 | 0/10 | 0/10 | 8/10 | 2/10 | 0/10 | 0/10 | 0/10 | 0/10 |
|  | MD | 0/10 | 0/10 | 0/10 | 8/10 | 2/10 | 0/10 | 0/10 | 0/10 | 0/10 |
|  | HD | 0/10 | 0/10 | 0/10 | 10/10 | 0/10 | 0/10 | 0/10 | 0/10 | 0/10 |
| 24 h | NC | 0/10 | 0/10 | 0/10 | 9/10 | 1/10 | 0/10 | 0/10 | 0/10 | 0/10 |
|  | PC | 0/10 | 0/10 | 0/10 | 8/10 | 2/10 | 0/10 | 0/10 | 0/10 | 0/10 |
|  | LD | 0/10 | 0/10 | 0/10 | 9/10 | 1/10 | 0/10 | 0/10 | 0/10 | 0/10 |
|  | MD | 0/10 | 0/10 | 0/10 | 10/10 | 0/10 | 0/10 | 0/10 | 0/10 | 0/10 |
|  | HD | 0/10 | 0/10 | 0/10 | 9/10 | 1/10 | 0/10 | 0/10 | 0/10 | 0/10 |

●Data are presented as frequency (affected animals/total animals per group). Compared with the negative control group, no significant differences were observed (*P* > 0.05). NC: negative control group; PC: positive control group; LD: low dose group, hKGF-2 eye drop groups (200 μg/ml); MD: medium dose group, hKGF-2 eye drop groups (500 μg/ml); HD: high dose group, hKGF-2 eye drop groups (1000 μg/ml).

**Supplementary Table 6** Effects of hKGF-2 on the CNS of rats: behavioral profiles

| **Parameters** | | Alertness | Finger approach | Head touch | Startle | Startle response | Positional passivity | Catalepsy | Visual placing | Tail pinch response | Body posture | Spontaneous locomotion | Ataxic gait | Rearing |
| --- | --- | --- | --- | --- | --- | --- | --- | --- | --- | --- | --- | --- | --- | --- |
| **Score range**  **/Reference** | | 0–8; 4 | 0–8; 4 | 0–8; 4 | 0–8; 4 | 0–8; 4 | 0–8; 4 | 0–4; 0 | 0–8; 4 | 0–8; 4 | 0–8; 4 | 0–8; 4 | 0–4; 0 | 0–8; 4 |
| **0 h** | **NC** | 4.0 ± 0.0 | 4.0 ± 0.0 | 4.0 ± 0.0 | 4.0 ± 0.0 | 4.0 ± 0.0 | 4.0 ± 0.0 | 0.0 ± 0.0 | 4.0 ± 0.0 | 4.0 ± 0.0 | 4.0 ± 0.0 | 4.0 ± 0.0 | 0.0 ± 0.0 | 4.6 ± 1.0 |
|  | **PC** | 4.0 ± 0.0 | 4.0 ± 0.0 | 4.0 ± 0.0 | 4.0 ± 0.0 | 4.0 ± 0.0 | 4.0 ± 0.0 | 0.0 ± 0.0 | 4.0 ± 0.0 | 4.0 ± 0.0 | 4.0 ± 0.0 | 4.0 ± 0.0 | 0.0 ± 0.0 | 4.8 ± 1.0 |
|  | **LD** | 4.0 ± 0.0 | 4.0 ± 0.0 | 4.0 ± 0.0 | 4.0 ± 0.0 | 4.0 ± 0.0 | 4.0 ± 0.0 | 0.0 ± 0.0 | 4.0 ± 0.0 | 4.0 ± 0.0 | 4.0 ± 0.0 | 4.0 ± 0.0 | 0.0 ± 0.0 | 4.8 ± 1.0 |
|  | **MD** | 4.0 ± 0.0 | 4.0 ± 0.0 | 4.0 ± 0.0 | 4.0 ± 0.0 | 4.0 ± 0.0 | 4.0 ± 0.0 | 0.0 ± 0.0 | 4.0 ± 0.0 | 4.0 ± 0.0 | 4.0 ± 0.0 | 4.0 ± 0.0 | 0.0 ± 0.0 | 4.8 ± 1.0 |
|  | **HD** | 4.0 ± 0.0 | 4.0 ± 0.0 | 4.0 ± 0.0 | 4.0 ± 0.0 | 4.0 ± 0.0 | 4.0 ± 0.0 | 0.0 ± 0.0 | 4.0 ± 0.0 | 4.0 ± 0.0 | 4.0 ± 0.0 | 4.0 ± 0.0 | 0.0 ± 0.0 | 4.6 ± 1.0 |
| **0.25 h** | **NC** | 4.0 ± 0.0 | 4.0 ± 0.0 | 4.0 ± 0.0 | 4.0 ± 0.0 | 4.0 ± 0.0 | 4.0 ± 0.0 | 0.0 ± 0.0 | 4.0 ± 0.0 | 4.0 ± 0.0 | 4.0 ± 0.0 | 4.0 ± 0.0 | 0.0 ± 0.0 | 4.4 ± 0.8 |
|  | **PC** | 0.0 ± 0.0^***^ | 0.0 ± 0.0^***^ | 0.0 ± 0.0^***^ | 0.0 ± 0.0^***^ | 0.0 ± 0.0^***^ | 0.0 ± 0.0^***^ | 3.1 ± 0.3^***^ | 2.0 ± 0.0^***^ | 3.0 ± 1.1^*^ | 0.0 ± 0.0^***^ | 0.0 ± 0.0^***^ | NE | 0.0 ± 0.0^***^ |
|  | **LD** | 4.0 ± 0.0 | 4.0 ± 0.0 | 4.0 ± 0.0 | 4.0 ± 0.0 | 4.0 ± 0.0 | 4.0 ± 0.0 | 0.0 ± 0.0 | 4.0 ± 0.0 | 4.0 ± 0.0 | 4.0 ± 0.0 | 4.0 ± 0.0 | 0.0 ± 0.0 | 4.4 ± 0.8 |
|  | **MD** | 4.0 ± 0.0 | 4.0 ± 0.0 | 4.0 ± 0.0 | 4.0 ± 0.0 | 4.0 ± 0.0 | 4.0 ± 0.0 | 0.0 ± 0.0 | 4.0 ± 0.0 | 4.0 ± 0.0 | 4.0 ± 0.0 | 4.0 ± 0.0 | 0.0 ± 0.0 | 4.6 ± 1.3 |
|  | **HD** | 4.0 ± 0.0 | 4.0 ± 0.0 | 4.0 ± 0.0 | 4.0 ± 0.0 | 4.0 ± 0.0 | 4.0 ± 0.0 | 0.0 ± 0.0 | 4.0 ± 0.0 | 4.0 ± 0.0 | 4.0 ± 0.0 | 4.0 ± 0.0 | 0.0 ± 0.0 | 4.4 ± 1.3 |
| **4 h** | **NC** | 4.0 ± 0.0 | 4.0 ± 0.0 | 4.0 ± 0.0 | 4.0 ± 0.0 | 4.0 ± 0.0 | 4.0 ± 0.0 | 0.0 ± 0.0 | 4.0 ± 0.0 | 4.0 ± 0.0 | 4.0 ± 0.0 | 4.0 ± 0.0 | 0.0 ± 0.0 | 4.6 ± 1.0 |
|  | **PC** | 0.8 ± 1.0^***^ | 0.8 ± 1.0^***^ | 1.0 ± 1.1^***^ | 1.4 ± 1.0^***^ | 4.0 ± 0.0 | 1.8 ± 1.8^**^ | 1.8 ± 0.4^**^ | 3.4 ± 1.0 | 2.6 ± 1.0^**^ | 0.2 ± 0.6^***^ | 1.4 ± 1.0^***^ | 2.0 ± 0.0^***^ | 0.0 ± 0.0^***^ |
|  | **LD** | 4.0 ± 0.0 | 4.0 ± 0.0 | 4.0 ± 0.0 | 4.0 ± 0.0 | 4.0 ± 0.0 | 4.0 ± 0.0 | 0.0 ± 0.0 | 4.0 ± 0.0 | 4.0 ± 0.0 | 4.0 ± 0.0 | 4.0 ± 0.0 | 0.0 ± 0.0 | 4.8 ± 1.0 |
|  | **MD** | 4.0 ± 0.0 | 4.0 ± 0.0 | 4.0 ± 0.0 | 4.0 ± 0.0 | 4.0 ± 0.0 | 4.0 ± 0.0 | 0.0 ± 0.0 | 4.0 ± 0.0 | 4.0 ± 0.0 | 4.0 ± 0.0 | 4.0 ± 0.0 | 0.0 ± 0.0 | 4.4 ± 1.3 |
|  | **HD** | 4.0 ± 0.0 | 4.0 ± 0.0 | 4.0 ± 0.0 | 4.0 ± 0.0 | 4.0 ± 0.0 | 4.0 ± 0.0 | 0.0 ± 0.0 | 4.0 ± 0.0 | 4.0 ± 0.0 | 4.0 ± 0.0 | 4.0 ± 0.0 | 0.0 ± 0.0 | 4.4 ± 1.3 |
| **24 h** | **NC** | 4.0 ± 0.0 | 4.0 ± 0.0 | 4.0 ± 0.0 | 4.0 ± 0.0 | 4.0 ± 0.0 | 4.0 ± 0.0 | 0.0 ± 0.0 | 4.0 ± 0.0 | 4.0 ± 0.0 | 4.0 ± 0.0 | 4.0 ± 0.0 | 0.0 ± 0.0 | 4.8 ± 1.0 |
|  | **PC** | 4.0 ± 0.0 | 4.0 ± 0.0 | 4.0 ± 0.0 | 4.0 ± 0.0 | 4.0 ± 0.0 | 4.0 ± 0.0 | 0.0 ± 0.0 | 4.0 ± 0.0 | 4.0 ± 0.0 | 4.0 ± 0.0 | 4.0 ± 0.0 | 0.0 ± 0.0 | 5.0 ± 1.1 |
|  | **LD** | 4.0 ± 0.0 | 4.0 ± 0.0 | 4.0 ± 0.0 | 4.0 ± 0.0 | 4.0 ± 0.0 | 4.0 ± 0.0 | 0.0 ± 0.0 | 4.0 ± 0.0 | 4.0 ± 0.0 | 4.0 ± 0.0 | 4.0 ± 0.0 | 0.0 ± 0.0 | 4.6 ± 1.0 |
|  | **MD** | 4.0 ± 0.0 | 4.0 ± 0.0 | 4.0 ± 0.0 | 4.0 ± 0.0 | 4.0 ± 0.0 | 4.0 ± 0.0 | 0.0 ± 0.0 | 4.0 ± 0.0 | 4.0 ± 0.0 | 4.0 ± 0.0 | 4.0 ± 0.0 | 0.0 ± 0.0 | 4.8 ± 1.4 |
|  | **HD** | 4.0 ± 0.0 | 4.0 ± 0.0 | 4.0 ± 0.0 | 4.0 ± 0.0 | 4.0 ± 0.0 | 4.0 ± 0.0 | 0.0 ± 0.0 | 4.0 ± 0.0 | 4.0 ± 0.0 | 4.0 ± 0.0 | 4.0 ± 0.0 | 0.0 ± 0.0 | 4.6 ± 1.0 |

● Except for NE (Not Evaluated), data are shown as the mean ± SD (n = 10). Compared with the negative control group, ^*^*P* < 0.05, ^**^*P* < 0.01, and ^***^*P* < 0.001. NC: negative control group; PC: positive control group; LD: low dose group, hKGF-2 eye drop groups (200 μg/ml); MD: medium dose group, hKGF-2 eye drop groups (500 μg/ml); HD: high dose group, hKGF-2 eye drop groups (1000 μg/ml).

**Supplementary Table 7 Effects of hKGF-2 on the CNS of rats: neurological examinations and autonomic signs**

| **Parameters** | | Grip strength | Body tone | Abdominal tone | Limb tone | Hypotonic gait | Corneal reflex | Pinna reflex | Hindlimb reflex | Righting reflex on the floor | Righting reflex in air | Urination | Defecation | Consistency of feces | Urine color |
| --- | --- | --- | --- | --- | --- | --- | --- | --- | --- | --- | --- | --- | --- | --- | --- |
| **Score range**  **/Reference** | | 0–8; 4 | 0–8; 4 | 0–8; 4 | 0–8; 4 | 0–4; 0 | 0–8; 4 | 0–8; 4 | 0–8; 4 | 0–4; 0 | 0–4; 0 | 0–8; 4 | 0–8; 4 | 0–8; 4 | 0–4; 0 |
| **0 h** | **NC** | 4.0 ± 0.0 | 4.0 ± 0.0 | 4.0 ± 0.0 | 4.0 ± 0.0 | 0.0 ± 0.0 | 4.0 ± 0.0 | 4.0 ± 0.0 | 4.0 ± 0.0 | 0.0 ± 0.0 | 0.0 ± 0.0 | 3.8 ± 0.6 | 3.0 ± 1.1 | 4.0 ± 0.0 | 0.0 ± 0.0 |
|  | **PC** | 4.0 ± 0.0 | 4.0 ± 0.0 | 4.0 ± 0.0 | 4.0 ± 0.0 | 0.0 ± 0.0 | 4.0 ± 0.0 | 4.0 ± 0.0 | 4.0 ± 0.0 | 0.0 ± 0.0 | 0.0 ± 0.0 | 3.4 ± 1.0 | 3.2 ± 1.0 | 4.0 ± 0.0 | 0.0 ± 0.0 |
|  | **LD** | 4.0 ± 0.0 | 4.0 ± 0.0 | 4.0 ± 0.0 | 4.0 ± 0.0 | 0.0 ± 0.0 | 4.0 ± 0.0 | 4.0 ± 0.0 | 4.0 ± 0.0 | 0.0 ± 0.0 | 0.0 ± 0.0 | 3.6 ± 1.3 | 3.2 ± 1.0 | 4.0 ± 0.0 | 0.0 ± 0.0 |
|  | **MD** | 4.0 ± 0.0 | 4.0 ± 0.0 | 4.0 ± 0.0 | 4.0 ± 0.0 | 0.0 ± 0.0 | 4.0 ± 0.0 | 4.0 ± 0.0 | 4.0 ± 0.0 | 0.0 ± 0.0 | 0.0 ± 0.0 | 3.6 ± 1.3 | 3.2 ± 1.0 | 4.0 ± 0.0 | 0.0 ± 0.0 |
|  | **HD** | 4.0 ± 0.0 | 4.0 ± 0.0 | 4.0 ± 0.0 | 4.0 ± 0.0 | 0.0 ± 0.0 | 4.0 ± 0.0 | 4.0 ± 0.0 | 4.0 ± 0.0 | 0.0 ± 0.0 | 0.0 ± 0.0 | 3.4 ± 1.0 | 3.4 ± 1.3 | 4.0 ± 0.0 | 0.0 ± 0.0 |
| **0.25 h** | **NC** | 4.0 ± 0.0 | 4.0 ± 0.0 | 4.0 ± 0.0 | 4.0 ± 0.0 | 0.0 ± 0.0 | 4.0 ± 0.0 | 4.0 ± 0.0 | 4.0 ± 0.0 | 0.0 ± 0.0 | 0.0 ± 0.0 | 3.0 ± 1.9 | 2.4 ± 1.6 | 4.0 ± 0.0 | 0.0 ± 0.0 |
|  | **PC** | 2.0 ± 0.0^***^ | 0.0 ± 0.0^***^ | 0.0 ± 0.0^***^ | 0.0 ± 0.0^***^ | NE | 3.2 ± 1.0^*^ | 3.0 ± 1.1^*^ | 2.0 ± 2.1* | 1.0 ± 1.1^*^ | 1.0 ± 1.1^*^ | 0.4 ± 0.8^**^ | 0.0 ± 0.0^**^ | NE | 4.0 ± 0.0^***^ |
|  | **LD** | 4.0 ± 0.0 | 4.0 ± 0.0 | 4.0 ± 0.0 | 4.0 ± 0.0 | 0.0 ± 0.0 | 4.0 ± 0.0 | 4.0 ± 0.0 | 4.0 ± 0.0 | 0.0 ± 0.0 | 0.0 ± 0.0 | 1.8 ± 1.1 | 1.4 ± 1.6 | 4.0 ± 0.0 | 0.0 ± 0.0 |
|  | **MD** | 4.0 ± 0.0 | 4.0 ± 0.0 | 4.0 ± 0.0 | 4.0 ± 0.0 | 0.0 ± 0.0 | 4.0 ± 0.0 | 4.0 ± 0.0 | 4.0 ± 0.0 | 0.0 ± 0.0 | 0.0 ± 0.0 | 2.6 ± 2.1 | 1.6 ± 2.1 | 4.0 ± 0.0 | 0.0 ± 0.0 |
|  | **HD** | 4.0 ± 0.0 | 4.0 ± 0.0 | 4.0 ± 0.0 | 4.0 ± 0.0 | 0.0 ± 0.0 | 4.0 ± 0.0 | 4.0 ± 0.0 | 4.0 ± 0.0 | 0.0 ± 0.0 | 0.0 ± 0.0 | 2.0 ± 1.3 | 1.6 ± 1.3 | 4.0 ± 0.0 | 0.0 ± 0.0 |
| **4 h** | **NC** | 4.0 ± 0.0 | 4.0 ± 0.0 | 4.0 ± 0.0 | 4.0 ± 0.0 | 0.0 ± 0.0 | 4.0 ± 0.0 | 4.0 ± 0.0 | 4.0 ± 0.0 | 0.0 ± 0.0 | 0.0 ± 0.0 | 3.0 ± 1.1 | 1.4 ± 1.0 | 4.0 ± 0.0 | 0.0 ± 0.0 |
|  | **PC** | 2.0 ± 0.0^***^ | 2.0 ± 0.0^***^ | 2.0 ± 0.0^***^ | 2.0 ± 0.0^***^ | 2.0 ± 0.0^***^ | 3.6 ± 0.8 | 2.6 ± 1.9^*^ | 2.0 ± 0.0^***^ | 0.4 ± 0.8 | 0.2 ± 0.4 | 3.6 ± 1.3 | 1.0 ± 1.7 | 3.2 ± 1.8 | 1.5 ± 2.1* |
|  | **LD** | 4.0 ± 0.0 | 4.0 ± 0.0 | 4.0 ± 0.0 | 4.0 ± 0.0 | 0.0 ± 0.0 | 4.0 ± 0.0 | 4.0 ± 0.0 | 4.0 ± 0.0 | 0.0 ± 0.0 | 0.0 ± 0.0 | 2.6 ± 1.3 | 2.0 ± 1.3 | 4.0 ± 0.0 | 0.0 ± 0.0 |
|  | **MD** | 4.0 ± 0.0 | 4.0 ± 0.0 | 4.0 ± 0.0 | 4.0 ± 0.0 | 0.0 ± 0.0 | 4.0 ± 0.0 | 4.0 ± 0.0 | 4.0 ± 0.0 | 0.0 ± 0.0 | 0.0 ± 0.0 | 3.2 ± 1.0 | 2.0 ± 1.3 | 4.0 ± 0.0 | 0.0 ± 0.0 |
|  | **HD** | 4.0 ± 0.0 | 4.0 ± 0.0 | 4.0 ± 0.0 | 4.0 ± 0.0 | 0.0 ± 0.0 | 4.0 ± 0.0 | 4.0 ± 0.0 | 4.0 ± 0.0 | 0.0 ± 0.0 | 0.0 ± 0.0 | 3.6 ± 0.8 | 2.2 ± 1.5 | 4.0 ± 0.0 | 0.0 ± 0.0 |
| **24 h** | **NC** | 4.0 ± 0.0 | 4.0 ± 0.0 | 4.0 ± 0.0 | 4.0 ± 0.0 | 0.0 ± 0.0 | 4.0 ± 0.0 | 4.0 ± 0.0 | 4.0 ± 0.0 | 0.0 ± 0.0 | 0.0 ± 0.0 | 3.2 ± 1.0 | 3.4 ± 1.0 | 4.0 ± 0.0 | 0.0 ± 0.0 |
|  | **PC** | 4.0 ± 0.0 | 4.0 ± 0.0 | 4.0 ± 0.0 | 4.0 ± 0.0 | 0.0 ± 0.0 | 4.0 ± 0.0 | 4.0 ± 0.0 | 4.0 ± 0.0 | 0.0 ± 0.0 | 0.0 ± 0.0 | 2.8 ± 1.4 | 3.2 ± 1.4 | 4.0 ± 0.0 | 0.0 ± 0.0 |
|  | **LD** | 4.0 ± 0.0 | 4.0 ± 0.0 | 4.0 ± 0.0 | 4.0 ± 0.0 | 0.0 ± 0.0 | 4.0 ± 0.0 | 4.0 ± 0.0 | 4.0 ± 0.0 | 0.0 ± 0.0 | 0.0 ± 0.0 | 3.2 ± 1.0 | 3.0 ± 1.4 | 4.0 ± 0.0 | 0.0 ± 0.0 |
|  | **MD** | 4.0 ± 0.0 | 4.0 ± 0.0 | 4.0 ± 0.0 | 4.0 ± 0.0 | 0.0 ± 0.0 | 4.0 ± 0.0 | 4.0 ± 0.0 | 4.0 ± 0.0 | 0.0 ± 0.0 | 0.0 ± 0.0 | 3.0 ± 1.1 | 3.2 ± 1.0 | 4.0 ± 0.0 | 0.0 ± 0.0 |
|  | **HD** | 4.0 ± 0.0 | 4.0 ± 0.0 | 4.0 ± 0.0 | 4.0 ± 0.0 | 0.0 ± 0.0 | 4.0 ± 0.0 | 4.0 ± 0.0 | 4.0 ± 0.0 | 0.0 ± 0.0 | 0.0 ± 0.0 | 3.0 ± 1.1 | 2.8 ± 1.4 | 4.0 ± 0.0 | 0.0 ± 0.0 |

● Except for NE (not evaluated), data are shown as the mean ± SD (n = 10). Compared with the negative control group, ^*^*P* < 0.05, ^**^*P* < 0.01, and ^***^*P* < 0.001. NC: Negative control group; PC: Positive control group; LD: Low dose group, hKGF-2 eye drop groups (200 μg/ml); MD: Medium dose group, hKGF-2 eye drop groups (500 μg/ml); HD: High dose group, hKGF-2 eye drop groups (1000 μg/ml).

**Supplementary Table 8** Effects of hKGF-2 on the rats' CNS: behavioral profiles and neurological examinations

| **Timepoints** | **Groups** | **Parameters of behavioral profiles** | | | | **Parameters of neurological examinations** | | | | | | |
| --- | --- | --- | --- | --- | --- | --- | --- | --- | --- | --- | --- | --- |
|  |  | Abnormal behavior | Grooming behavior | Aggression / Irritability | Abnormal vocalization | Convulsion | Seizure | Tremor | Respiration | Heart rate | Skin color | Cyanosis |
| **0 h** | **NC** | 0.0 ± 0.0 | 0.0 ± 0.0 | 0.0 ± 0.0 | 0.0 ± 0.0 | 0.0 ± 0.0 | 0.0 ± 0.0 | 0.0 ± 0.0 | 4.0 ± 0.0 | 0.0 ± 0.0 | 4.0 ± 0.0 | 0.0 ± 0.0 |
|  | **PC** | 0.0 ± 0.0 | 0.0 ± 0.0 | 0.0 ± 0.0 | 0.0 ± 0.0 | 0.0 ± 0.0 | 0.0 ± 0.0 | 0.0 ± 0.0 | 4.0 ± 0.0 | 0.0 ± 0.0 | 4.0 ± 0.0 | 0.0 ± 0.0 |
|  | **LD** | 0.0 ± 0.0 | 0.0 ± 0.0 | 0.0 ± 0.0 | 0.0 ± 0.0 | 0.0 ± 0.0 | 0.0 ± 0.0 | 0.0 ± 0.0 | 4.0 ± 0.0 | 0.0 ± 0.0 | 4.0 ± 0.0 | 0.0 ± 0.0 |
|  | **MD** | 0.0 ± 0.0 | 0.0 ± 0.0 | 0.0 ± 0.0 | 0.0 ± 0.0 | 0.0 ± 0.0 | 0.0 ± 0.0 | 0.0 ± 0.0 | 4.0 ± 0.0 | 0.0 ± 0.0 | 4.0 ± 0.0 | 0.0 ± 0.0 |
|  | **HD** | 0.0 ± 0.0 | 0.0 ± 0.0 | 0.0 ± 0.0 | 0.0 ± 0.0 | 0.0 ± 0.0 | 0.0 ± 0.0 | 0.0 ± 0.0 | 4.0 ± 0.0 | 0.0 ± 0.0 | 4.0 ± 0.0 | 0.0 ± 0.0 |
| **0.25 h** | **NC** | 0.0 ± 0.0 | 0.0 ± 0.0 | 0.0 ± 0.0 | 0.0 ± 0.0 | 0.0 ± 0.0 | 0.0 ± 0.0 | 0.0 ± 0.0 | 4.0 ± 0.0 | 0.0 ± 0.0 | 4.0 ± 0.0 | 0.0 ± 0.0 |
|  | **PC** | 0.0 ± 0.0 | 0.0 ± 0.0 | 0.0 ± 0.0 | 0.0 ± 0.0 | 0.0 ± 0.0 | 0.0 ± 0.0 | 0.0 ± 0.0 | 4.0 ± 0.0 | 0.0 ± 0.0 | 4.0 ± 0.0 | 0.0 ± 0.0 |
|  | **LD** | 0.0 ± 0.0 | 0.0 ± 0.0 | 0.0 ± 0.0 | 0.0 ± 0.0 | 0.0 ± 0.0 | 0.0 ± 0.0 | 0.0 ± 0.0 | 4.0 ± 0.0 | 0.0 ± 0.0 | 4.0 ± 0.0 | 0.0 ± 0.0 |
|  | **MD** | 0.0 ± 0.0 | 0.0 ± 0.0 | 0.0 ± 0.0 | 0.0 ± 0.0 | 0.0 ± 0.0 | 0.0 ± 0.0 | 0.0 ± 0.0 | 4.0 ± 0.0 | 0.0 ± 0.0 | 4.0 ± 0.0 | 0.0 ± 0.0 |
|  | **HD** | 0.0 ± 0.0 | 0.0 ± 0.0 | 0.0 ± 0.0 | 0.0 ± 0.0 | 0.0 ± 0.0 | 0.0 ± 0.0 | 0.0 ± 0.0 | 4.0 ± 0.0 | 0.0 ± 0.0 | 4.0 ± 0.0 | 0.0 ± 0.0 |
| **4 h** | **NC** | 0.0 ± 0.0 | 0.0 ± 0.0 | 0.0 ± 0.0 | 0.0 ± 0.0 | 0.0 ± 0.0 | 0.0 ± 0.0 | 0.0 ± 0.0 | 4.0 ± 0.0 | 0.0 ± 0.0 | 4.0 ± 0.0 | 0.0 ± 0.0 |
|  | **PC** | 0.0 ± 0.0 | 0.0 ± 0.0 | 0.0 ± 0.0 | 0.0 ± 0.0 | 0.0 ± 0.0 | 0.0 ± 0.0 | 0.0 ± 0.0 | 4.0 ± 0.0 | 0.0 ± 0.0 | 4.0 ± 0.0 | 0.0 ± 0.0 |
|  | **LD** | 0.0 ± 0.0 | 0.0 ± 0.0 | 0.0 ± 0.0 | 0.0 ± 0.0 | 0.0 ± 0.0 | 0.0 ± 0.0 | 0.0 ± 0.0 | 4.0 ± 0.0 | 0.0 ± 0.0 | 4.0 ± 0.0 | 0.0 ± 0.0 |
|  | **MD** | 0.0 ± 0.0 | 0.0 ± 0.0 | 0.0 ± 0.0 | 0.0 ± 0.0 | 0.0 ± 0.0 | 0.0 ± 0.0 | 0.0 ± 0.0 | 4.0 ± 0.0 | 0.0 ± 0.0 | 4.0 ± 0.0 | 0.0 ± 0.0 |
|  | **HD** | 0.0 ± 0.0 | 0.0 ± 0.0 | 0.0 ± 0.0 | 0.0 ± 0.0 | 0.0 ± 0.0 | 0.0 ± 0.0 | 0.0 ± 0.0 | 4.0 ± 0.0 | 0.0 ± 0.0 | 4.0 ± 0.0 | 0.0 ± 0.0 |
| **24 h** | **NC** | 0.0 ± 0.0 | 0.0 ± 0.0 | 0.0 ± 0.0 | 0.0 ± 0.0 | 0.0 ± 0.0 | 0.0 ± 0.0 | 0.0 ± 0.0 | 4.0 ± 0.0 | 0.0 ± 0.0 | 4.0 ± 0.0 | 0.0 ± 0.0 |
|  | **PC** | 0.0 ± 0.0 | 0.0 ± 0.0 | 0.0 ± 0.0 | 0.0 ± 0.0 | 0.0 ± 0.0 | 0.0 ± 0.0 | 0.0 ± 0.0 | 4.0 ± 0.0 | 0.0 ± 0.0 | 4.0 ± 0.0 | 0.0 ± 0.0 |
|  | **LD** | 0.0 ± 0.0 | 0.0 ± 0.0 | 0.0 ± 0.0 | 0.0 ± 0.0 | 0.0 ± 0.0 | 0.0 ± 0.0 | 0.0 ± 0.0 | 4.0 ± 0.0 | 0.0 ± 0.0 | 4.0 ± 0.0 | 0.0 ± 0.0 |
|  | **MD** | 0.0 ± 0.0 | 0.0 ± 0.0 | 0.0 ± 0.0 | 0.0 ± 0.0 | 0.0 ± 0.0 | 0.0 ± 0.0 | 0.0 ± 0.0 | 4.0 ± 0.0 | 0.0 ± 0.0 | 4.0 ± 0.0 | 0.0 ± 0.0 |
|  | **HD** | 0.0 ± 0.0 | 0.0 ± 0.0 | 0.0 ± 0.0 | 0.0 ± 0.0 | 0.0 ± 0.0 | 0.0 ± 0.0 | 0.0 ± 0.0 | 4.0 ± 0.0 | 0.0 ± 0.0 | 4.0 ± 0.0 | 0.0 ± 0.0 |

●Data are shown as the mean ± SD (n = 10). Compared with the negative control group, no significant differences were observed (*P* > 0.05). NC: negative control group; PC: positive control group; LD: low dose group, hKGF-2 eye drop groups (200 μg/ml); MD: medium dose group, hKGF-2 eye drop groups (500 μg/ml); HD: high dose group, hKGF-2 eye drop groups (1000 μg/ml).

**Supplementary Table 9** Effects of hKGF-2 on the rats' CNS: autonomic signs

| **Timepoints** | **Groups** | **Parameters of autonomic signs** | | | | | | | | | |
| --- | --- | --- | --- | --- | --- | --- | --- | --- | --- | --- | --- |
|  |  | Writhing | Tail position | Piloerection | Palpebral  closure | Exophthalmos | Pupil size | Lacrimation | Chromodacryorrhea | Salivation | Mortality |
| **0 h** | **NC** | 0.0 ± 0.0 | 0.0 ± 0.0 | 0.0 ± 0.0 | 0.0 ± 0.0 | 0.0 ± 0.0 | 4.0 ± 0.0 | 4.0 ± 0.0 | 0.0 ± 0.0 | 4.0 ± 0.0 | 0/10 |
|  | **PC** | 0.0 ± 0.0 | 0.0 ± 0.0 | 0.0 ± 0.0 | 0.0 ± 0.0 | 0.0 ± 0.0 | 4.0 ± 0.0 | 4.0 ± 0.0 | 0.0 ± 0.0 | 4.0 ± 0.0 | 0/10 |
|  | **LD** | 0.0 ± 0.0 | 0.0 ± 0.0 | 0.0 ± 0.0 | 0.0 ± 0.0 | 0.0 ± 0.0 | 4.0 ± 0.0 | 4.0 ± 0.0 | 0.0 ± 0.0 | 4.0 ± 0.0 | 0/10 |
|  | **MD** | 0.0 ± 0.0 | 0.0 ± 0.0 | 0.0 ± 0.0 | 0.0 ± 0.0 | 0.0 ± 0.0 | 4.0 ± 0.0 | 4.0 ± 0.0 | 0.0 ± 0.0 | 4.0 ± 0.0 | 0/10 |
|  | **HD** | 0.0 ± 0.0 | 0.0 ± 0.0 | 0.0 ± 0.0 | 0.0 ± 0.0 | 0.0 ± 0.0 | 4.0 ± 0.0 | 4.0 ± 0.0 | 0.0 ± 0.0 | 4.0 ± 0.0 | 0/10 |
| **0.25 h** | **NC** | 0.0 ± 0.0 | 0.0 ± 0.0 | 0.0 ± 0.0 | 0.0 ± 0.0 | 0.0 ± 0.0 | 4.0 ± 0.0 | 4.0 ± 0.0 | 0.0 ± 0.0 | 4.0 ± 0.0 | 0/10 |
|  | **PC** | 0.0 ± 0.0 | 0.0 ± 0.0 | 0.0 ± 0.0 | 0.0 ± 0.0 | 0.0 ± 0.0 | 4.0 ± 0.0 | 4.0 ± 0.0 | 0.0 ± 0.0 | 4.0 ± 0.0 | 0/10 |
|  | **LD** | 0.0 ± 0.0 | 0.0 ± 0.0 | 0.0 ± 0.0 | 0.0 ± 0.0 | 0.0 ± 0.0 | 4.0 ± 0.0 | 4.0 ± 0.0 | 0.0 ± 0.0 | 4.0 ± 0.0 | 0/10 |
|  | **MD** | 0.0 ± 0.0 | 0.0 ± 0.0 | 0.0 ± 0.0 | 0.0 ± 0.0 | 0.0 ± 0.0 | 4.0 ± 0.0 | 4.0 ± 0.0 | 0.0 ± 0.0 | 4.0 ± 0.0 | 0/10 |
|  | **HD** | 0.0 ± 0.0 | 0.0 ± 0.0 | 0.0 ± 0.0 | 0.0 ± 0.0 | 0.0 ± 0.0 | 4.0 ± 0.0 | 4.0 ± 0.0 | 0.0 ± 0.0 | 4.0 ± 0.0 | 0/10 |
| **4 h** | **NC** | 0.0 ± 0.0 | 0.0 ± 0.0 | 0.0 ± 0.0 | 0.0 ± 0.0 | 0.0 ± 0.0 | 4.0 ± 0.0 | 4.0 ± 0.0 | 0.0 ± 0.0 | 4.0 ± 0.0 | 0/10 |
|  | **PC** | 0.0 ± 0.0 | 0.0 ± 0.0 | 0.0 ± 0.0 | 0.0 ± 0.0 | 0.0 ± 0.0 | 4.0 ± 0.0 | 4.0 ± 0.0 | 0.2 ± 0.4 | 4.0 ± 0.0 | 0/10 |
|  | **LD** | 0.0 ± 0.0 | 0.0 ± 0.0 | 0.0 ± 0.0 | 0.0 ± 0.0 | 0.0 ± 0.0 | 4.0 ± 0.0 | 4.0 ± 0.0 | 0.0 ± 0.0 | 4.0 ± 0.0 | 0/10 |
|  | **MD** | 0.0 ± 0.0 | 0.0 ± 0.0 | 0.0 ± 0.0 | 0.0 ± 0.0 | 0.0 ± 0.0 | 4.0 ± 0.0 | 4.0 ± 0.0 | 0.0 ± 0.0 | 4.0 ± 0.0 | 0/10 |
|  | **HD** | 0.0 ± 0.0 | 0.0 ± 0.0 | 0.0 ± 0.0 | 0.0 ± 0.0 | 0.0 ± 0.0 | 4.0 ± 0.0 | 4.0 ± 0.0 | 0.0 ± 0.0 | 4.0 ± 0.0 | 0/10 |
| **24 h** | **NC** | 0.0 ± 0.0 | 0.0 ± 0.0 | 0.0 ± 0.0 | 0.0 ± 0.0 | 0.0 ± 0.0 | 4.0 ± 0.0 | 4.0 ± 0.0 | 0.0 ± 0.0 | 4.0 ± 0.0 | 0/10 |
|  | **PC** | 0.0 ± 0.0 | 0.0 ± 0.0 | 0.0 ± 0.0 | 0.0 ± 0.0 | 0.0 ± 0.0 | 4.0 ± 0.0 | 4.0 ± 0.0 | 0.0 ± 0.0 | 4.0 ± 0.0 | 0/10 |
|  | **LD** | 0.0 ± 0.0 | 0.0 ± 0.0 | 0.0 ± 0.0 | 0.0 ± 0.0 | 0.0 ± 0.0 | 4.0 ± 0.0 | 4.0 ± 0.0 | 0.0 ± 0.0 | 4.0 ± 0.0 | 0/10 |
|  | **MD** | 0.0 ± 0.0 | 0.0 ± 0.0 | 0.0 ± 0.0 | 0.0 ± 0.0 | 0.0 ± 0.0 | 4.0 ± 0.0 | 4.0 ± 0.0 | 0.0 ± 0.0 | 4.0 ± 0.0 | 0/10 |
|  | **HD** | 0.0 ± 0.0 | 0.0 ± 0.0 | 0.0 ± 0.0 | 0.0 ± 0.0 | 0.0 ± 0.0 | 4.0 ± 0.0 | 4.0 ± 0.0 | 0.0 ± 0.0 | 4.0 ± 0.0 | 0/10 |

●Data are shown as the mean ± SD (n = 10), except for mortality, which is expressed as incidence (affected/total). Compared with the negative control group, no significant differences were observed (*P* > 0.05). NC: negative control group; PC: positive control group; LD: low dose group, hKGF-2 eye drop groups (200 μg/ml); MD: medium dose group, hKGF-2 eye drop groups (500 μg/ml); HD: high dose group, hKGF-2 eye drop groups (1000 μg/ml).

**Supplementary Table 10**

Effects of hKGF-2 on the rats' CNS: body temperature

| **Timepoints** | **NC** | **PC** | **LD** | **MD** | **HD** |
| --- | --- | --- | --- | --- | --- |
| 0 h | 38.4 ± 0.6 | 38.3 ± 0.3 | 38.2 ± 0.3 | 38.2 ± 0.3 | 38.1 ± 0.3 |
| 0.25 h | 38.5 ± 0.3 | 37.5 ± 1.0** | 38.7 ± 0.4 | 38.6 ± 0.4 | 38.6 ± 0.2 |
| 4 h | 37.5 ± 0.7 | 35.7 ± 1.1** | 37.2 ± 0.3 | 37.7 ± 0.4 | 37.3 ± 0.2 |
| 24 h | 37.8 ± 0.4 | 37.9 ± 0.4 | 37.8 ± 0.7 | 38.0 ± 0.4 | 37.8 ± 0.5 |

●Data are shown as the mean ± SD (n = 10), Compared with the negative control group, ^*^*P* < 0.05, ^**^ *P* <0.01, and ^***^*P* < 0.001. NC: negative control group; PC: positive control group; LD: low dose group, hKGF-2 eye drop groups (200 μg/ml); MD: medium dose group, hKGF-2 eye drop groups (500 μg/ml); HD: high dose group, hKGF-2 eye drop groups (1000 μg/ml).
